# Analysis of miRNAs and their target genes associated with lipid metabolism in duck liver

**DOI:** 10.1038/srep27418

**Published:** 2016-06-08

**Authors:** Jun He, Weiqun Wang, Lizhi Lu, Yong Tian, Dong Niu, Jindong Ren, Liyan Dong, Siwei Sun, Yan Zhao, Li Chen, Jianliang Shen, Xiuhong Li

**Affiliations:** 1 Department of Animal Science, Wenzhou Vocational College of Science & Technology, Wenzhou 325006, P.R. China; 2 Institute of Animal Husbandry and Veterinary Science, Zhejiang Academy Agricultural Sciences, Hangzhou 310021, P.R. China; 3 Department of Human Nutrition, Kansas State University, Manhattan, Kansas, 66506, USA; 4 College of Animal Science, Zhejiang University, Hangzhou 310021, P.R. China; 5 Zhejiang Zhuowang Agriculture Sci-Tech Limited Co., Huzhou 313014, P.R. China

## Abstract

Fat character is an important index in duck culture that linked to local flavor, feed cost and fat intake for costumers. Since the regulation networks in duck lipid metabolism had not been reported very clearly, we aimed to explore the potential miRNA-mRNA pairs and their regulatory roles in duck lipid metabolism. Here, Cherry-Valley ducks were selected and treated with/without 5% oil added in feed for 2 weeks, and then fat content determination was performed on. The data showed that the fat contents and the fatty acid ratios of C17:1 and C18:2 were up-regulated in livers of oil-added ducks, while the C12:0 ratio was down-regulated. Then 21 differential miRNAs, including 10 novel miRNAs, were obtain from the livers by sequencing, and 73 target genes involved in lipid metabolic processes of these miRNAs were found, which constituted 316 miRNA-mRNA pairs. Two miRNA-mRNA pairs including one novel miRNA and one known miRNA, N-miR-16020-FASN and gga-miR-144-ELOVL6, were selected to validate the miRNA-mRNA negative relation. And the results showed that N-mir-16020 and gga-miR-144 could respectively bind the 3′-UTRs of FASN and ELOVL6 to control their expressions. This study provides new sights and useful information for future research on regulation network in duck lipid metabolism.

MicroRNAs (miRNA), about 18–25 nucleotides in length, are small noncoding RNAs which possess a regulatory role in mRNA translation. Over 30 years ago, the first miRNA was discovered in the nematode Caenorhabditis elegans with the identification of the developmental regulator lin-4^1^, which is originally considered to be a conventional protein coding gene. While the Ruvkun and Ambros labs startlingly discovered that lin-4 encoded for a 22-nucleotide-long regulatory RNA instead for a protein, which could base pair with the mRNA of lin-14, another gene in the C. elegans developmental network, and control its expression^2^,^3^. Subsequently, many thousands of miRNAs were successively discovered in various organisms, for example, in human genome there had been 2588 miRNAs detected and annotated^4^. During miRNA biogenesis, a long primary miRNA (pri-miRNA) is firstly transcribed from the genome, fold into hairpins with two arms (5′ and 3′), and then undergo cleavage to generate a ∼70–100 nt long, hairpin-containing precursor miRNA (pre-miRNA)^5^,^6^, which would be further cleaved into an ∼18–25 nt miRNA duplex^7^. One strand of the duplex will be chosen as the mature miRNA to be assembled into the miRNA-induced silencing complex (miRISC)^8^. As translational inhibition, the miRISC will bind target mRNAs to facilitate their degradations^6^,^9^. While the other strand would be directly degraded^10^,^11^. In animals, miRNAs control the expressions of their target genes by a complementary binding between the seed regions (ranging from 2 to 8 nt) of miRNAs and 3′-UTRs of the target mRNAs. However, in plants, miRNAs target mRNAs through near-complete base pairing^6^,^12^,^13^.

It has been demonstrated that miRNAs regulate different biological processes such as apoptosis, organismal development, cell proliferation, tissue differentiation and regeneration^6^,^14^,^15^,^16^,^17^,^18^. Moreover, miRNAs also play crucial roles in many diseases, including cancer. For example, miR-17-92 and miR-21 were found elevated, while other miRNA families, including let-7 and miR-34 families, were frequently detected down-regulated in cancer^19^. The miR-17-92 cluster was reported elevated in many cancer types, especially in several leukemias and lymphomas^20^. Besides cancer, miRNAs are also linked to biological metabolisms, such as glucose metabolism, lipid metabolism and so on. miR-375 has been detected highly expressed in pancreatic islet cells and proved to control a large number of genes that involved in islet cell proliferation^21^,^22^. Let-7 and the miR-103/107 family have also been validated functionally linked to glucose metabolism^23^,^24^,^25^. miR-122, a liver-specific and liver-enriched miRNA, is the first validated miRNA that regulates lipid metabolism. Data showed that deletion of miR-122 could lead to decrease of serum triglyceride (TG) and cholesterol levels^26^,^27^,^28^. miR-33a/33b were investigated capable of binding the mRNAs of Sterol Regulatory Binding Element Factor (SREBP) genes and control their expressions. And SREBPs were considered as key transcriptional regulators in lipid metabolism. They had been proved to mediate many genes involved in fatty-acid, cholesterol biosynthesis and intake, phospholipids and TG productions, and so on^29^,^30^. Besides miR-122 and miR-33a/33b, there are still a certain number of miRNAs reported to be related to lipid metabolism, such as miR-27^31^,^32^,^33^,^34^, miR-378^35^,^36^, miR-370^37^ and so on. Most reports on the regulatory roles of miRNAs in lipid metabolism focused on mammals, but few data described which miRNAs mediated lipid metabolism and their mechanisms in poultry, especially in duck. In fact, lipid metabolism in duck is very important for not only the customers but also the raisers, because the fat characters of duck are directly linked to people’s intakes of TG and cholesterol, flavors of meat and egg, and the cost of feeding. Lipid metabolism in duck mainly locates in liver^38^,^39^. Thus, researches on lipid metabolism-related miRNAs and their mechanisms in duck liver would be meaningful.

In this study, feed with different fat contents was prepared and fed to ducks for 2 weeks, and then the miRNAs in all liver tissues were extracted and detected by high throughput sequencing. After differential expression analysis and miRNA-mRNA target prediction, lipid metabolism related miRNAs were collected and then validated. As a result, miRNAs that regulated lipid metabolism in duck liver were preliminarily found and their target genes were predicted. It should be helpful to explore and build the regulatory networks of the lipid metabolism in duck and other poultry.

## Results

### Differential fat characters analysis

The crude fat contents in livers of experimental group (EG) were detected significantly higher than those of control group (CG) (Table 1), promoted by 9.7%. To better analyze the fat characters, each constituent fatty acid content was also determined. As the statistical results showed that compared to CG ducks, the contents of C17:1 (Ginkgolic acid) and C18:2 (Linoleic acid) of EG ducks were significantly higher (*P* < 0.05), while the content of C12:0 (Lauric acid) was significantly lower (*P* < 0.05).

### miRNA expression profiles in duck liver

**Figure 1:**
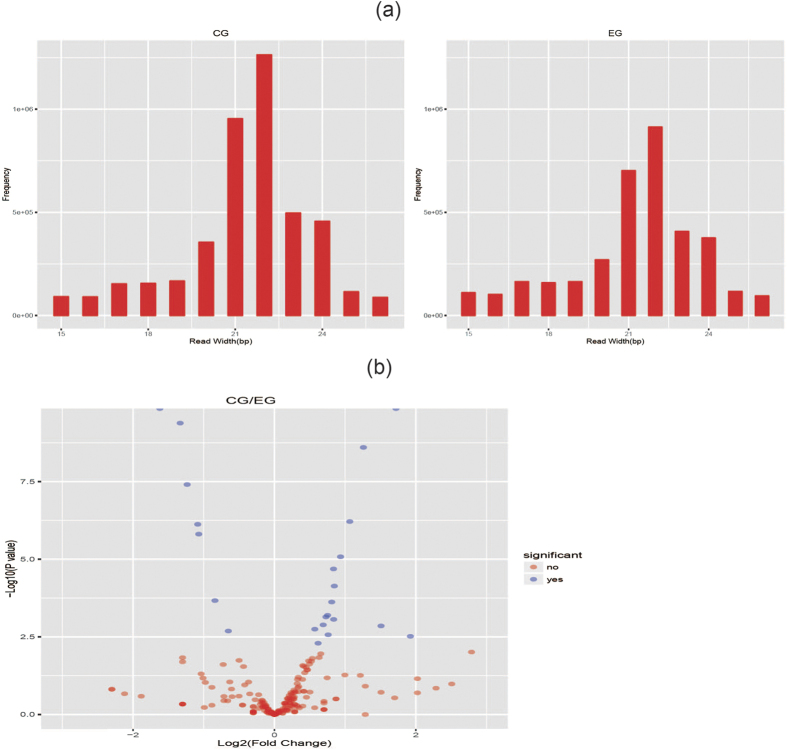
Read width frequency distributions (**a**) and volcano plot (**b**) of miRNAs from CG and EG livers. CG and EG represent control group and experimental group, respectively. This also applies to the following Figures and Tables.

**Figure 2:**
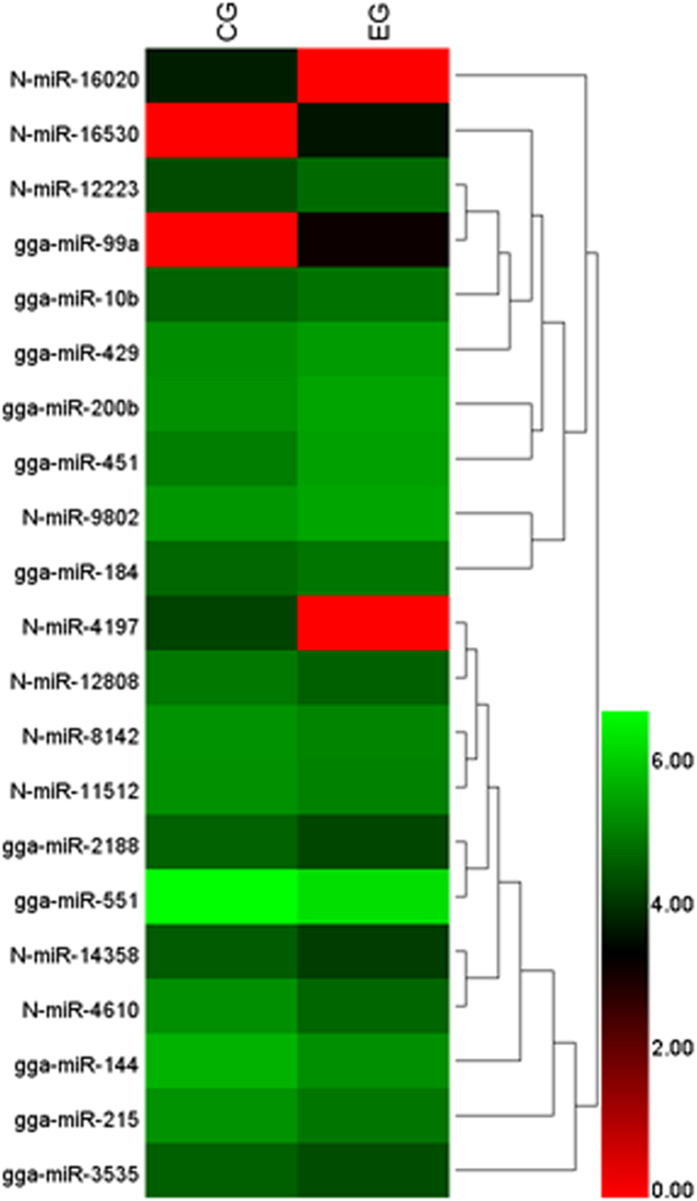
Heatmap of differentially expressed miRNAs in CG and EG livers. The prefix “N-” means predicted novel miRNAs.

By sequencing analysis, the information of a total of 557 miRNAs from all liver samples was obtained, and their frequencies (Fig. 1a), volcano plot (Fig. 1b), Scatter plot (see Supplementary Fig. S1) and expression density distributions (see Supplementary Fig. S2) in the two groups were analyzed, respectively. Furthermore, as the statistical results indicated that compared to CG livers, 21 miNRAs were found differentially expressed, among which 9 miRNAs were up-regulated while the other 12 miRNAs were down-regulated in EG livers. The differential expressions of the 21 miRNAs in CG and EG were presented in Fig. 2. By comparison with the known miRNA database in miRBase, 11 of the 21 differentially expressed miRNAs were known while the other 10 were predicted novel miRNAs, whose mature sequences were displayed in Table 2.

### miRNA target analysis

**Figure 3:**
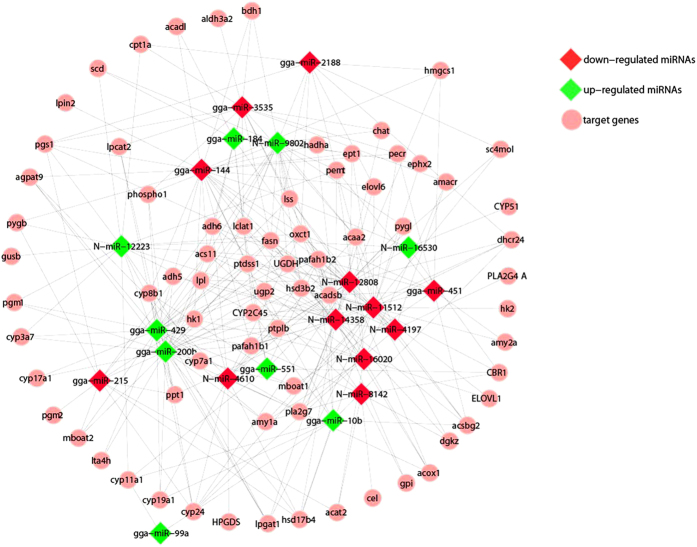
Regulation networks of the differentially expressed miRNAs and their predicted target genes in duck lipid metabolism.

**Figure 4:**
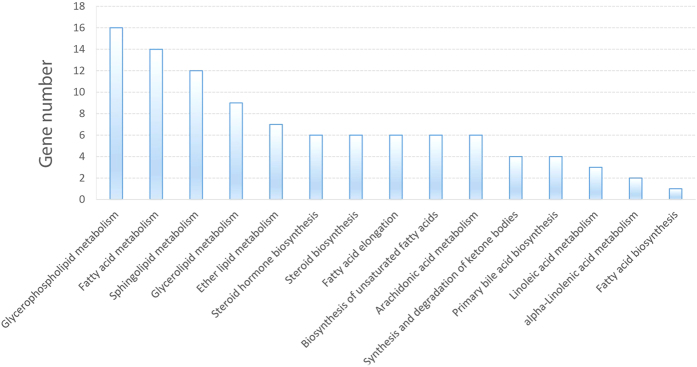
Target gene Numbers of the differential miRNAs in duck lipid metabolic processes.

To characterize the regulations of miRNAs on lipid metabolism in duck, miRNA target prediction, GO enrichment and KEGG pathway analysis were performed. As a result, 316 most scored potential miRNA-mRNA pairs were obtained between the 21 differentially expressed miRNAs and 73 genes that involved in 15 lipid metabolic processes. The network of the 21 miRNAs and the 73 predicted target genes were constructed using Cytoscape software and shown in Fig. 3. The distribution of the 73 target genes in different metabolic processes was shown in Fig. 4.

### Validation of two miRNA/mRNA negative regulation pairs

**Figure 5:**
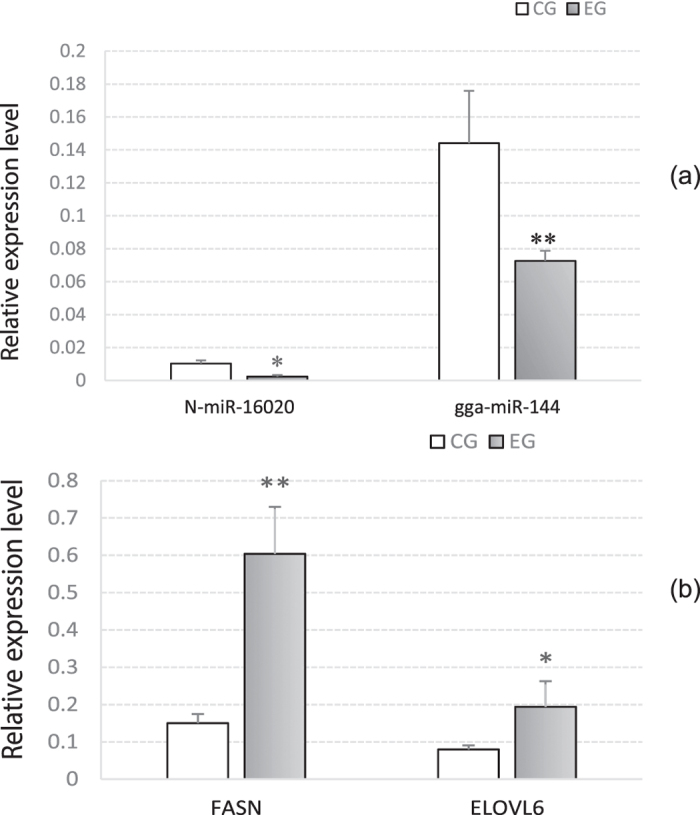
Relative expression levels of N-miR-16020 and gga-miR-144 (**a**) and their chosen target genes FASN and ELOVL6 (**b**) in CG and EG. Expression levels of the two miRNAs and their chosen target genes were determined by qRT-PCR in the CG and EG liver samples, normalized to those of U6 and β-actin respectively, and then shown as mean ± SD. Each transfection was performed in triplicates. *P < 0.05, **P < 0.01.

**Figure 6:**
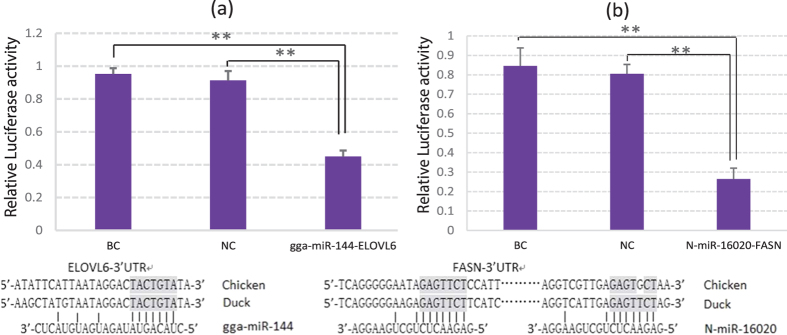
Dual-Luciferase reporter system validations of gga-miR-144-ELOVL6 (**a**) and N-miR-16020-FASN (**b**). Recombined Dual –Luciferase reporter vectors with 3′-UTR of target genes were cotransfected with miRNAs, while cotransfection with negative mimics and transfection without mimics were settled as negative control (NC) and blank control (BC), respectively. Each transfection was performed in 6 replicates. The firefly: *Renilla* luciferase activities were calculated and shown as mean ± SD. *P < 0.05, **P < 0.01. The information of sequence analysis between miRNA seed regions and target 3′-UTRs was placed at the bottom.

N-miR-16020 and gga-miR-144 were selected to perform the validation analysis. Primers were designed to detect their expressions in CG and EG livers. And the qRT-PCR results indicated that compared to CG, both expressions of N-miR-16020 (*P* < 0.05) and gga-miR-144 (*P* < 0.01) were down-regulated in EG (Fig. 5a). Among the predicted target genes of gga-miR-144 and N-miR-16020, Elongation of very Long chain fatty acids protein 6 (ELOVL6) and Fatty acid synthase (FASN) were respectively chosen to perform Dual-Luciferase reporter system analysis. The recombined reporter vectors with the 3′-UTRs of ELOVL6/FASN were cotransfected into duck hepatocytes with gga-miR-144/N-miR-16020 mimics, respectively. Transfections with negative mimics and without mimics were settled as negative control (NC) and blank control (BC), respectively. Each transfection was settled for 6 replicates. Data of duck hepatocyte isolation and identification could be found as Supplementary Fig. S3. After determination and statistical analysis, the firefly: *Renilla* luciferase activities of the two miRNA-target validations were both significantly lowered by gga-miR-144/N-miR-16020 mimics (*P* < 0.01) compared to NC and BC (Fig. 6a,b). Furthermore, the relative expression levels of the two chosen target genes in CG and EG livers, FASN and ELOVL6, were also determined up-regulated by 303% (*P* < 0.01) and 144% (*P* < 0.05), respectively (Fig. 5b).

## Discussion

Duck provides important parts for people’s diet, such as meat and eggs. However, duck products with high fat content would cause many related diseases that threating people’s health, such as obesity, angiocardiopathy, hepatic adipose infiltration and so on. Besides, excessive lipid metabolism in duck will reduce its nutritional value and lower down feed conversion efficiency, which leads to more cost of feed. miRNAs have been reported to play fundamental roles in regulations of various biological processes, including lipid metabolism. Since few data were available on miRNAs involved in duck lipid metabolism which mainly locates in liver tissue, an analysis of lipid metabolism-related miRNAs in duck liver was performed on.

In this study, the feed of the experimental ducks was added in with 5% oil. And after 2 weeks, the fat contents of the livers (3.153 ± 0.049%^a^) were detected significantly higher than those of the control (2.875 ± 0.035%^b^), which indicates that the lipid synthesis in the experimental ducks represented more active. Then the differential miRNAs analysis was performed on, and 21 differentially-expressed, lipid metabolism-related miRNAs were discovered, among which 11 miRNAs were matched known in miRBase database while the other 10 miRNAs were predicted novel. These miRNAs were considered to play regulatory roles in duck lipid metabolism, and similar results had been reported in other animals. For example, miR-451 was detected significantly down-regulated (*P* < 0.05) in mice livers when fed with atherogenic diet^40^, and miR-451 was also found involved in human non-alcoholic fatty liver (NAFLD)^41^. A high-throughput Solexa sequencing approach was adopted to identify the miRNAs differentially expressed in large White pigs (lean type pig) and Meishan pigs (Chinese indigenous fatty pig)^42^, as the results showed that the expression level of miR-215 in Large White pigs was significantly higher (*P* < 0.01) than that in Meishan pigs. Several lipid metabolism-related diseases were proved that could be caused by the abnormal expression of miR-144^43^,^44^. These data were consistent with the results in this study.

By sequence matching and energy stability assessment, 316 pairs of miRNA-target gene were obtain in this study, containing 21 differential miRNAs and 73 lipid metabolism-related genes. To validate the predictions, two miRNA-target gene pairs were selected to test their regulatory relations, gga-miR-144-ELOVL6 and N-miR-16020-FASN, including one known miRNA and one predicted novel miRNA. Firstly, the expression levels of the two miRNAs in the livers were detected, and the qRT-PCR analysis indicated that both the two miRNAs were significantly down-regulated (*P* < 0.05) in EG livers (Fig. 5a), which was consistent with the miRNA sequencing data. Then Dual-Luciferase reporter system analysis was performed on to validate the regulations of the selected miRNA-target gene pairs, and the results represented that both the firefly: Renilla luciferase activity values were significantly lowered down (*P* < 0.01) by cotransfection with miRNA mimics, compared to negative control and blank control. These data indicated that the applied miRNA-target gene prediction analysis in this article was reliable to some extent. Furthermore, the expression levels of FASN and ELOVL6 genes were also determined in the liver samples by qRT-PCT, as the data shown in Fig. 5b, the expressions of the two genes were both significantly promoted in EG livers (*P* < 0.05), which was in accordance with the expression changes of N-miR-16020 and gga-miR-144. The up-regulated expressions of FASN and ELOVL6 might explain the higher contents of crude fat, C17:1, C18:2 and lower content of C12:0 in EG livers. FASN is a crucial multifunctional enzyme in lipogenesis and deposition^45^,^46^, and ELOVL6 was demonstrated to elongate C12-C16 to longer fatty acids, especially C18^47^,^48^,^49^. By the way, since each miRNA was predicted to regulate the expressions of a number of target genes, which were also predicted regulated by many miRNAs^12^, the expression changes of FASN and ELOVL6 genes in EG were probably the outcome of co-regulation by different miRNAs, including the up-regulated miRNAs. The promoted expressions of these miRNAs could be caused by a feedback regulation of their target genes or some other mechanisms, which needed to be further researched.

Fat characters of duck are directly related to both culturing cost and people’s health. Therefore, it is necessary to understand the regulation networks in duck lipid metabolism. In this research, a number of lipid metabolism-related miRNAs were obtained, and their target genes in lipid metabolic processes were predicted and preliminarily validated. In conclusion, this study may provide useful information and new objects for future research on lipid metabolism in duck.

## Materials and Methods

### Ethics statement

All experimental protocols were approved by Animal Ethics committee of Zhejiang University (Hangzhou, China). The methods were conducted in accordance with the Guidelines for Experimental Animals established by the Ministry of Science and Technology (Beijing, China).

### Animal and tissue collection

Two groups (n = 5) of Cherry-Valley ducks at the age of 10 weeks with the same genetic background were selected as control group (CG) and experimental group (EG) in this study. Both groups of ducks were watered ad libitum and provided with the same amount of feed each day, in addition, the feed of EG was added in with 5% oil (Animal oil∶vegetable oil, 1:1). After 2 weeks, liver samples of all individuals were immediately frozen into liquid nitrogen and lately stored at −70 °C. All animals in this experiment were slaughtered following ethical standards.

### Determination of fat content

The fat contents of liver samples were determined following Soxhelt extraction method, in which petroleum ether was used as solvent. Furthermore, the fatty acid ratios of the fat in liver samples were also detected. All liver samples were directly analyzed by Gas Chromatography-Mass Spectrometer (GC-MS) on a fused silica column (HP-5MS, 30 m × 0.25 mm × 0.25 μm, length × ID × film thickness). High-purity Helium (99.9995%, 0.7 mL/min) was used as carrier gas to flush pyrolytic compounds into the GC-MS system (6890N GC/5973MS, Agilent). The column temperature was elevated from 100 °C to 220 °C (6 °C/min), held for 13 min, and then elevated again to 260 °C (10 °C/min), held for 15 min. The mass spectrometer was operated in SCAN model with ionization energy of 70 eV (EI), ion source at 230 °C and quadrupole at 150 °C. The compounds were then compared to NIST98 to detect the structures, and the contents of each fatty acid were determined by calculating the peak areas.

### RNA extraction

Total RNA of each sample was extracted with TRIzol reagent (Invitrogen, USA) following the manufacturer’s protocols, and then treated with RQ1 DNase (Promega, USA) to remove DNA. The quality and quantity of the extracted RNA were detected by measuring the absorbance at 260 nm/280 nm using NanoDrop 2000 (Thermo, USA). And then the RNA integrity was further verified by 1.5% agarose gel electrophoresis.

### miRNA sequencing and statistical analysis

The extracted RNAs were ligated to 3′ and 5′ adaptors sequentially, reverse transcribed and PCR amplified. The entire library was then tested by 10% native PAGE gel electrophoresis, and the bands corresponding to microRNA insertion were cut and eluted. After purification, the small RNA libraries were quantified with Qubit Fluorometer (Invitrogen, USA) and then used for cluster generation and 50 nt single end sequencing analysis with Illumina HiSeq2000 system (Illumina, USA) according to the manufacturer’s instructions. The raw digital-quality data were analyzed by masking adaptor sequences and removing the contaminated reads, after which the clean reads were collected and aligned with the chicken reference genome and duck 3′UTR data (NCBI). The clean reads were compared with the Rfam database to filter the known non-miRNA reads, such as rRNA, snoRNA, snRNA, HACA-box, ribozyme, CRISPR, IRES, scaRNA and so on, and then compared with the known miRNA database in miRBase to identify mature miRNAs or pre-miRNAs. In addition, novel miRNAs were predicted through the algorithm of miRDeep^50^, and RNA-fold was used to analyze their structures. The numbers of miRNA reads were normalized by Fragments Per Kilobase of transcript per Million fragments mapped (FPKM)^51^. The differential significance was identified by the EdgeR program^52^ at adj. *P* ≤ 0.01.

### miRNA target predictions and analysis

miRNA-3′-UTR sequence matching and energy stability assessment were analyzed by Miranda algorithm to predict potential target genes of the obtained lipid metabolism related miRNAs. And then GO Term and KEGG pathway analyses of these genes were performed.

### qRT-PCR validation

miRNAs and mRNAs were reverse transcribed to cDNA with the miRcute miRNA First-Strand cDNA Synthesis Kit (TIANGEN, Beijing, China) and PrimeScript RT reagent Kit with gDNA Eraser (Takara, Japan), respectively. U6 RNA and β-actin were chosen as endogenous internal controls, and the relative expression levels were calculated as 2^−ΔCt^ (ΔCt = Ct_*target*_ − Ct_*control*_). qRT-PCR analyses were performed on ABI 7300 system (Applied Biosystems, Foster City, CA) using SYBR Green Realtime PCR Mix (Takara, Japan) in triplicate for each reaction.

### Dual-Luciferase reporter system validation

Primer pairs with different restriction sites added in the forward and reverse primers were designed to amplify the 3′-UTRs (containing miRNA binding sites) of the chosen target genes in duck, and then recombined to pmirGLO Dual-Luciferase miRNA Target Expression Vector (Promega, USA) following the manufacturer’s introductions, which was pre-treated with the same restriction endonucleases. After verification by restriction and sequencing, the recombined vector was cotransfected into isolated Cherry-Valley duck hepatocytes with miRNA mimics/negative control (Shanghai GenePhama, China). After 48 h of cell culture, the luciferase activities were detected with Dual-Luciferase Reporter Assay System (Promega, USA) by Smart Line TL Tube Luminometer (Titertek Berthold, Germany) according to the manufacturers’ protocols. Each cotransfection was performed in 6 replicates.

## Additional Information

**How to cite this article**: He, J. *et al.* Analysis of miRNAs and their target genes associated with lipid metabolism in duck liver. *Sci. Rep.* **6**, 27418; doi: 10.1038/srep27418 (2016).

## Supplementary Material

Supplementary Information

## Figures and Tables

**Table 1:** Fat contents in liver tissues of control group (CG) and experimental group (EG).

Fatty acids	CG	EG	*P*_value
**C12:0**	**2.440 ± 0.022%**^**a**^	**2.270 ± 0.043%**^**b**^	**0.0163**
C13:0	0.323 ± 0.012%	0.300 ± 0.014%	1.1561
C14:0	2.091 ± 0.016%	2.107 ± 0.026%	0.4959
C14:1	0.927 ± 0.012%	0.937 ± 0.012%	0.4676
C15:0	0.077 ± 0.009%	0.053 ± 0.005%	0.0534
C15:1	0.420 ± 0.008%	0.433 ± 0.005%	0.1335
C16:0	22.397 ± 0.034%	22.420 ± 0.024%	0.4791
C16:1	3.101 ± 0.022%	3.057 ± 0.012%	0.0858
C17:0	1.801 ± 0.012%	1.797 ± 0.017%	0.5421
**C17:1**	**0.113 ± 0.005%**^**a**^	**0.127 ± 0.005%**^**b**^	**0.0474**
C18:0	16.568 ± 0.031%	16.643 ± 0.017%	0.0519
C18:1	21.884 ± 0.122%	21.913 ± 0.028%	0.7708
**C18:2**	**13.228 ± 0.026%**^**a**^	**13.317 ± 0.021%**^**b**^	**0.0208**
C18:3	0.027 ± 0.005%	0.030 ± 0.006%	0.4227
C20:0	0.013 ± 0.005%	0.020 ± 0.009%	0.1835
C20:1	0.036 ± 0.009%	0.043 ± 0.005%	0.4381
C20:2	1.313 ± 0.025%	1.343 ± 0.009%	0.2251
C20:3	11.691 ± 0.041%	11.593 ± 0.012%	0.0681
C20:5	0.023 ± 0.005%	0.020 ± 0.003%	0.4227
C22:0	1.407 ± 0.012%	1.437 ± 0.012%	0.0739
C22:1	0.077 ± 0.009%	0.087 ± 0.005%	0.2739
C22:3	0.010 ± 0.003%	0.013 ± 0.005%	0.4226
C22:5	0.023 ± 0.005%	0.030 ± 0.06%	0.1835
C22:6	0.010 ± 0.001%	0.010 ± 0.002%	0.5355
Total	100%	100%	
**Crude fat**	**2.875 ± 0.035%**^**a**^	**3.153 ± 0.049%**^**b**^	**0.0152**

The contents of each fatty acid and the crude fat were calculated as ratios (means ± SD) to total fatty acids and liver weights, respectively. The values in fold indicate significant differences at *P* < 0.05.

**Table 2:** Expression changes and mature sequences of the 21 differentially expressed miRNAs.

miRNAs	Expression change	Mature sequences (5′→3′)
gga-miR-99a	Up	AACCCGUAGAUCCGAUCUUGUG
gga-miR-10b	Up	UACCCUGUAGAACCGAAUUUGU
gga-miR-184	Up	UGGACGGAGAACUGAUAAGGGU
gga-miR-200b	Up	UAAUACUGCCUGGUAAUGAUGAU
gga-miR-429	Up	UAAUACUGUCUGGUAAUGCCGU
gga-miR-551	Up	GCGACCCAUACUUGGUUUCAG
gga-miR-215	Down	AUGACCUAUGAAUUGACAGAC
gga-miR-451	Down	AAACCGUUACCAUUACUGAGUUU
gga-miR-2188	Down	AAGGUCCAACCUCACAUGUCCU
gga-miR-3535	Down	GGAUAUGAUGACUGAUUAUCUGAAA
gga-miR-144	Down	CUACAGUAUAGAUGAUGUACUC
N-miR-16020	Down	GAGAACUCUGCUGAAGGA
N-miR-16530	Up	CUGGCGAUGAUGACACACU
N-miR-9802	Up	UGGGAUGAAGCACUGAAGC
N-miR-12223	Up	CGGCUGAGAUCUGAUAGC
N-miR-4197	Down	UGAUCUCAUCCUGGGGCU
N-miR-12808	Down	GUCCAAGCCGGACGGACU
N-miR-14358	Down	GCAGACUCACUCUGUACUGAACU
N-miR-4610	Down	CUAGAUGAUCUUAGAAACU
N-miR-8142	Down	UGUUGUAGAUCUGGCACA
N-miR-11512	Down	CACUGCUGCAGUGACUCCC

Predicted novel miRNAs in the table were randomly named with the prefix “N-”. Expression changes represented the changes in EG that compared to CG.
