# Supplementary material for: Analysis of miRNAs and their target genes associated with lipid metabolism in duck liver
Source: Sci Rep. 2016 Jun 8;6:27418. doi: 10.1038/srep27418 (PMC4897641; doi:10.1038/srep27418)
Supplement: Supplementary Information [file srep27418-s1.pdf]

Analysis of miRNAs and their target genes associated with lipid metabolism in duck liver

Jun He[1,2], Weiqun Wang[3], Lizhi Lu[2], Yong Tian[2], Dong Niu[4], Jindong Ren[2,5], Liyan Dong[1,2], Siwei Sun[1], Yan Zhao[1], Guoqin Li[2], Jianliang Shen[5], Xiuhong Li[1*]

[1]Department of Animal Science, Wenzhou Vocational College of Science & Technology, Wenzhou 325006, P. R. China. [2]Institute of Animal Husbandry and Veterinary Science, Zhejiang Academy Agricultural Sciences, Hangzhou 310021, P. R. China. [3]Department of Human Nutrition, Kansas State University, Manhattan, Kansas, 66506, USA. [4]College of Animal Science, Zhejiang University, Hangzhou 310021, P. R. China. [5]Zhejiang Zhuowang Agriculture Sci-Tech Limited Co., Huzhou 313014, P. R. China. *Correspondence and requests for materials should be addressed to X. L. (email: lixiuhong_wky@163.com)

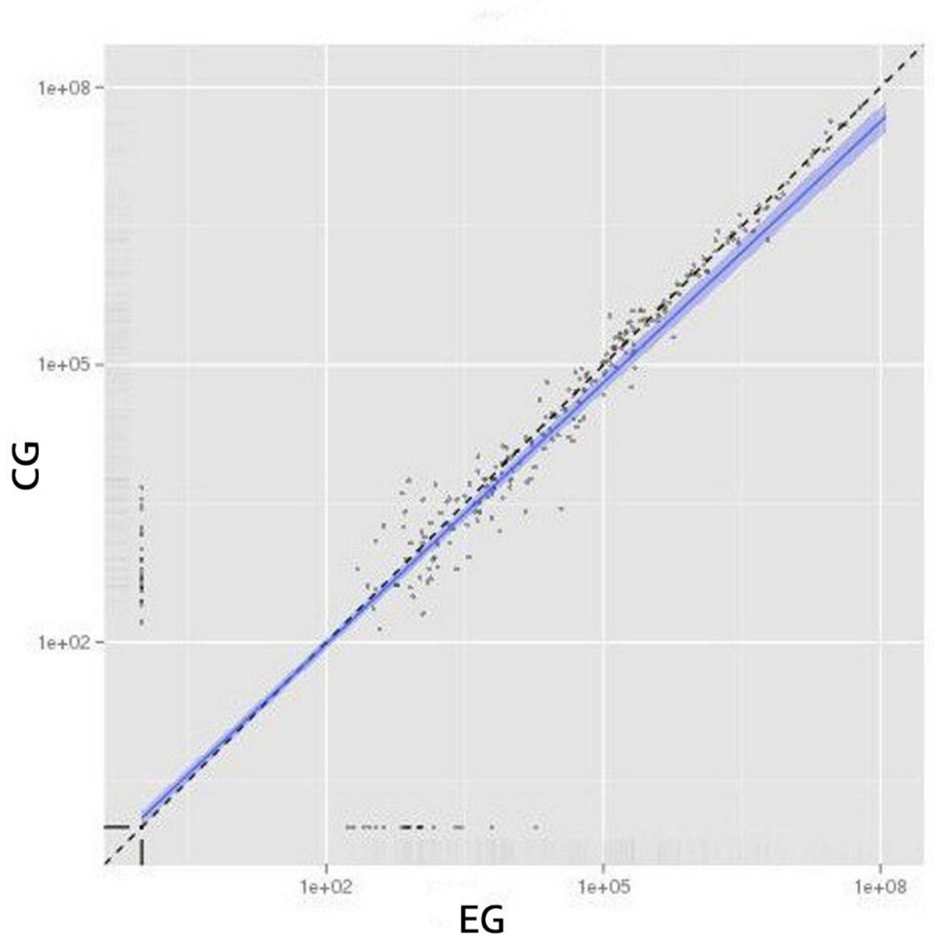

Supplementary Figure S1. Scatter plot (b) of the miRNA expression comparisons between control group (CG) and experimental group (EG). Differences were considered significant at adj. $P \leq 0.01$.

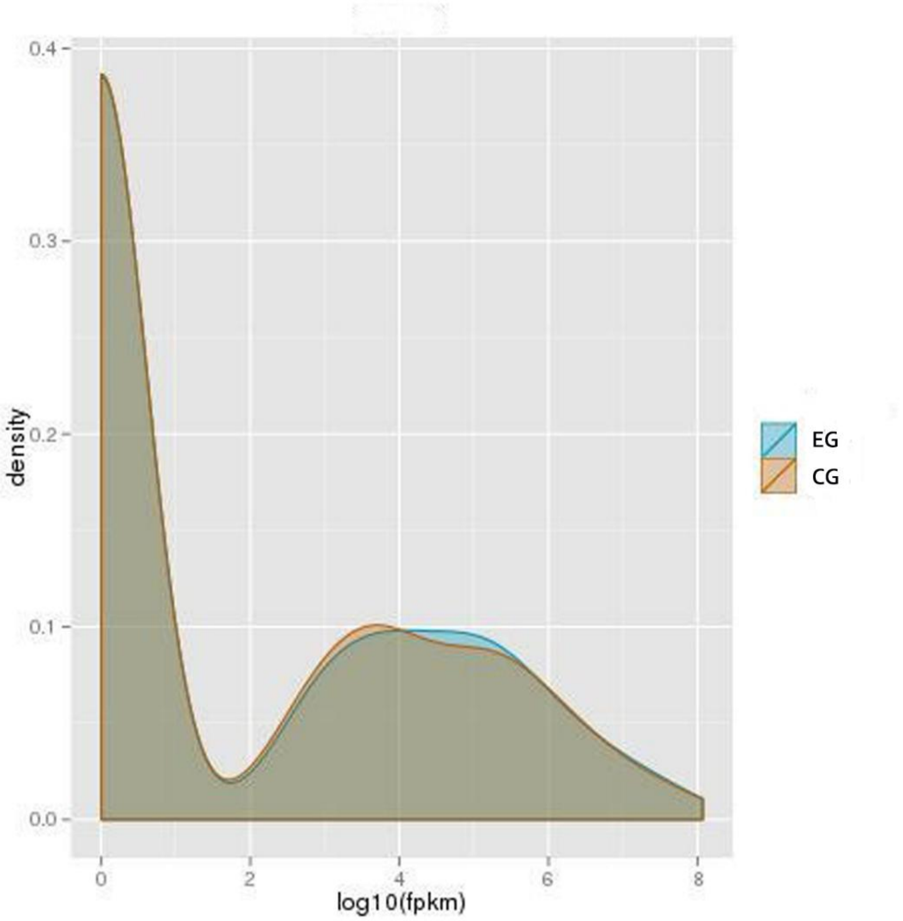

Supplementary Figure S2. miRNA expression density distributions in CG and EG livers

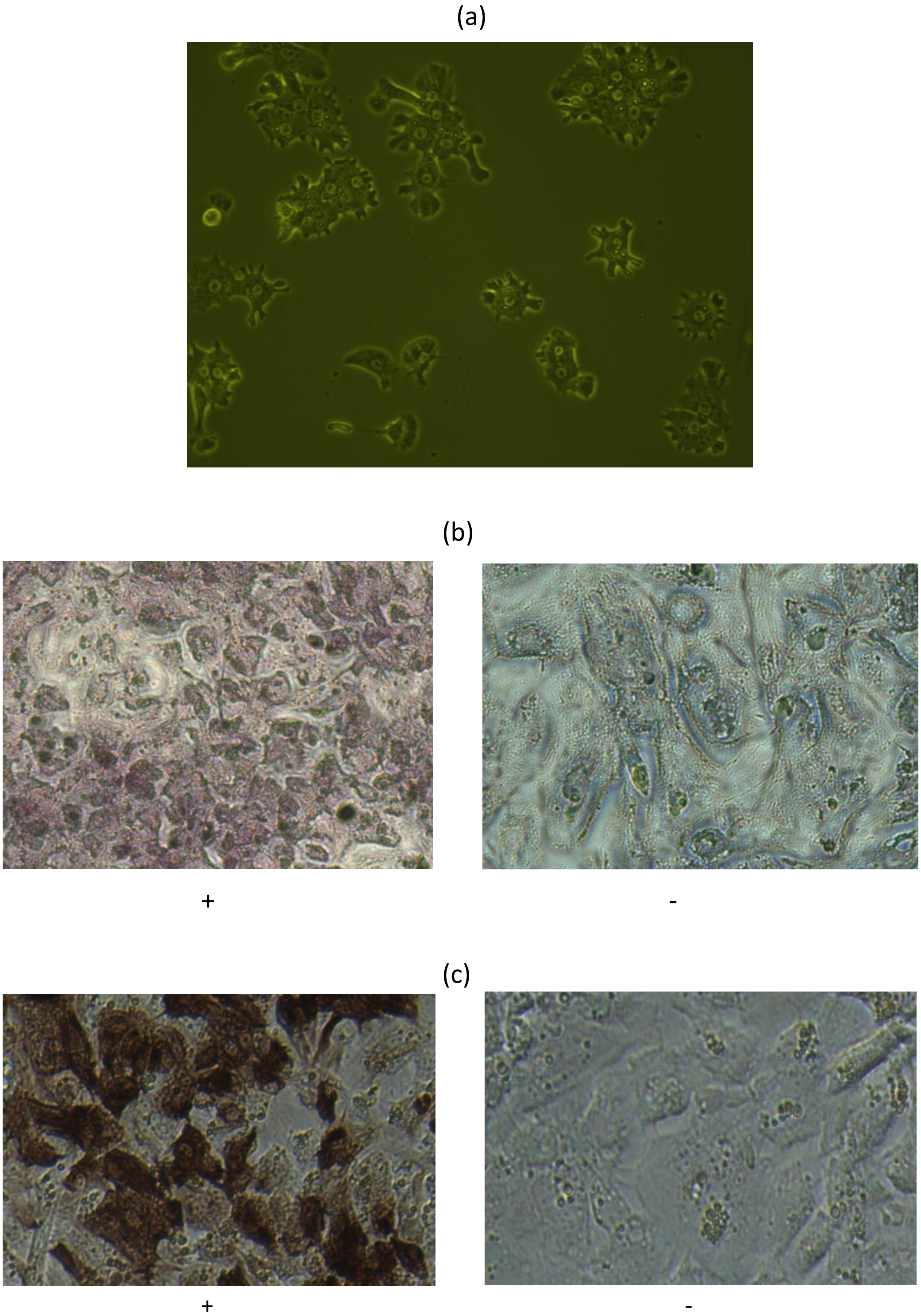

Supplementary Figure S3. Isolation (a) and identification (b and c) of duck hepatocytes. Hepatocytes were isolated for Cherry-Valley duck (age of 1 week) following the method of Seglen (1976). Periodic acid-Schiff stain (b) and immunohistochemistry (c) with Anti-Cytokeratin 18 antibody (Abcam, UK) were performed to identify the isolated duck hepatocytes following the producers' protocols.
